# Chemotactic invasion in deep soft tissue by *Vibrio vulnificus* is essential for the progression of necrotic lesions

**DOI:** 10.1080/21505594.2020.1782707

**Published:** 2020-06-27

**Authors:** Kohei Yamazaki, Takashige Kashimoto, Takehiro Kado, Yukihiro Akeda, Kazuki Yoshioka, Toshio Kodama, Mai Yamamoto, Masashi Okamura, Tsutomu Kakuda, Shunji Ueno

**Affiliations:** a Laboratory of Veterinary Public Health, School of Veterinary Medicine, Kitasato University, Aomori, Japan; b Division of Infection Control and Prevention, Osaka University Hospital, Osaka, Japan; c Laboratory of Veterinary Anatomy, School of Veterinary Medicine, Kitasato University, Aomori, Japan; d Department of Bacterial Infections, International Research Center for Infectious Diseases, Research Institute for Microbial Diseases, Osaka, Japan; e Laboratory of Nutritional Science, Okayama Prefectural University, Okayama, Japan; f Laboratory of Zoonosis, School of Veterinary Medicine, Kitasato University, Aomori, Japan; g Laboratory of Animal Hygiene, School of Veterinary Medicine, Kitasato University, Aomori, Japan

**Keywords:** *Vibrio vulnificus*, necrotizing soft tissue infection, pathogenic factor, chemotaxis, mouse model, in vivo imaging

## Abstract

Necrotizing soft tissue infections (NSTI) progress to severe necrosis and result in fatal sepsis within a short time. *Vibrio vulnificus* is a causative agent and can spread from the initial infection site through soft tissue finally to the systemic circulation of the host. The motility and chemotaxis of this bacterium are essential for proliferation and lethality in a murine model of the infection, but their role in pathogenicity has not been characterized. In this study, we revealed the roles of motility and chemotaxis during the process of *V. vulnificus* infection. We compared a nonmotile mutant and two nonchemotactic mutants with their parent strain (WT) with regard to bacterial spread using an in vivo imaging system (IVIS) and invasion by detection of bacteria from the muscle and spleen of a murine infection model. WT rapidly spread throughout the infected thigh and invaded deep muscle causing severe tissue damage. The detection rate in the systemic circulation and the lethality were high. On the other hand, the nonmotile mutant stayed at the inoculation site, and the nonchemotactic mutants spread only slowly through the soft tissue of the infected thigh. Detection in the systemic circulation, the degree of tissue damage, and the lethality of nonchemotactic mutants were significantly reduced in mice compared with WT. This study demonstrated that chemotaxis is essential for invasion from the infection site to the deep and distant tissues and the main pathogenic factor for the rapid progression leading to sepsis in *V. vulnificus* NSTI.

## Introduction

Necrotizing soft-tissue infections (NSTIs) such as severe soft tissue necrosis and sepsis are the most dramatically progressing bacterial infections. *Vibrio vulnificus* is a major causative bacterium of NSTI [1–4]. Due to its ability to expand rapidly within a short time and cause severe necrotic lesions, prompt diagnosis, aggressive surgical debridement, and systemic antibacterial therapy are required. *V. vulinificus* NSTI occurs via exposure through an open wound (wound infection) or via the ingestion of raw seafood (primary septicemia) [4–9]. This pathogen lives in warmish, brackish water regions, and wound infections are increasing owing to rising seawater temperatures caused by climate change [5,8,9]. Sudden onset of NSTI, rapid necrosis progression, and limited knowledge of the pathogenic mechanisms remain as issues for establishing therapeutic strategies. *V. vulnificus* has a capsule and Rtx (repeats in toxin) to resist phagocytosis and promote colonization, and an iron acquisition system to efficiently proliferate [10–14]. In addition, cytolysin VVH [11] and metalloprotease VvpE [15,16] are secreted by *V. vulnificus* and are thought to play some role in infection. However, the factors contributing to the rapid expansion of the necrotic lesion have not been clarified.

Flagellar-based motility and chemotaxis were identified as the essential factors to establish wound infection of *V. vulnificus* in our previous study [17]. The flagellum is one of the main structures for motile bacteria and a major antigen for recognition by the host cells through the toll-like receptor [18,19], and its mutant shows a striking phenotype both *in vitro* and *in vivo*. In fact, a decrease in lethality is seen in nonmotile mutants of *V. vulnificus* in mice. Some studies have shown that flagellar-based motility is necessary for its virulence in murine sepsis models by intraperitoneal injection or intravascular injection [20–22]. These reports showed that flagellar-based motility is essential for lethality in systemic infection, but it was not precisely clear when and where the chemotaxis is required during the course of NSTI from the original local infection to death.

Bacterial chemotaxis is a two-component system consisting of a membrane-bound histidine kinase that senses a specific environmental stimulus and a corresponding response regulator that transmits the signals to the flagellar rotator [23–25]. These systems allow bacteria to sense the concentration gradient of chemical substances and move toward an attractant or away from a repellent by regulating the direction of flagellar rotation. Nevertheless, there is little evidence of a role for chemotaxis in pathogenesis even in other bacterial pathogens, let alone for *V. vulnificus*. Uropathogenic *E. coli* (UPEC) has chemotaxis toward urine and can proliferate in the bladder [26]. Chemotactic receptors of *H. pylori* sense acids, and the bacterium migrates to the gastric gland by chemotaxis and can proliferate in the stomach [27]. In these study on bacterial chemotaxis and pathogenicity, nonchemotactic mutant Δ*cheY* was used to find a preferred colonization site where the mutant cannot invade due to lacking chemotaxis [17,27,28]. Interestingly, Butler and Camilli found a niche, where *Vibrio cholerae* invades and proliferates, by using two phenotypes; mutants of Δ*cheY* and a point mutant of CheY. The Δ*cheY* lacks the CheY activation that is essential for chemotaxis regulation and is characterized by smooth bias swimming [29,30]. The point mutation of *cheY* can stabilize CheY phosphorylation (activation) characterized by a tumble bias [28,31]. They found that the smooth swimming as Δ*cheY* was essential for invasion into the intestinal crypt by comparing with the tumble biased mutant [28]. The chemotaxis contributes to the underlying pathogenesis of disease by facilitating pathogen invasion into their preferred niche, where they can then proliferate, colonize, and form the focus of infection.

To date, chemotaxis is believed to be important for the rapid spread of bacteria and the rapid expansion of the necrotic lesion, but there have been no studies on the role of chemotaxis in the pathogenesis of NSTI. In this study, we used a wound infection model and compared two nonchemotactic mutants with their parent strain (WT). The nonchemotactic mutants had significantly reduced virulence and lethality. This was due to a reduction in the efficient systemic spread of the bacterium from the local infection site based on chemotaxis. Our data highlighted the importance of chemotaxis in the severity of NSTI.

## Materials and methods

### Ethics statement

All animal studies were carried out in strict accordance with the Guidelines for Animal Experimentation of the Japanese Association for Laboratory Animal Science (JALAS). The animal experimentation protocol was approved by the president of Kitasato University based on the judgment of the Institutional Animal Care and Use Committee of Kitasato University and by the Animal Care and Use Committee of Osaka University (Approval No. 15–156 and No. 17–268).

### Bacteria

*V. vulnificus* clinical isolated strain CMCP6 was cultured aerobically in Luria-Bertani (LB) broth or on LB agar at 37°C. When required, the medium was supplemented with rifampicin (50 µg/mL), chloramphenicol (10 µg/mL), or ampicillin (100 µg/mL) for *V. vulnificus* to maintain the pXen plasmid and the pACYC plasmid (Fig. S1).

### Mutant construction and complementation

The Δ*pomA* and Δ*cheY* deletion mutants were constructed as described previously [17]. Briefly, the *pomA* gene and the *cheY* gene were amplified and cloned into the suicide vector pYAK1, retaining *sacB*. pYAK1-*pomA*KO and pYAK1-*cheY*KO were introduced into *V. vulnificus* CMCP6. The bacteria retaining pYAK1-*pomA*KO or pYAK1-*cheY*KO was cultured in LB broth containing 20% sucrose following the standard *sacB*-assisted allelic exchange method. Mutants were confirmed by PCR to detect expected changes in size at the *pomA* locus or the *cheY* locus. The Δ*cheY* mutant was complemented with the full‐length *cheY* gene carried by pACYC. The complementation strain p*cheY* was cultured in LB containing 10 μg/mL chloramphenicol.

### Random mutagenesis

Randomly mutated *cheY* was created in a 50 µl volume containing template genome DNA, Ex Taq polymerase (TaKaRa Bio, Shiga, Japan), 10× PCR buffer, dNTPs, the primers *cheY* Fw (GGATCCTTGAATAAAAACATGAAGATCCTTATT) and *cheY* down (CTCGAGTTATAAACGTTCAAAAATTTTATCTAG), and MnCl_2_ (final 200 µM) [32]. The resulting DNA fragment was cloned into the pGEM-T Easy vector and subcloned into pACYC using the *Bam*HI and *Xho*I restriction sites. Tumble biased mutants were selected by swarming assay with LB plate containing 0.3% agar. The mutated sites of *cheY* were determined using the Fasmac sequencing service (Atsugi, Japan). The randomly mutated strain Q93R was cultured in LB containing 10 μg/mL chloramphenicol.

### Swarming assay

Bacterial suspensions were inoculated on LB containing 0.3% agar. Plates were incubated at 37°C for 12 h.

### Tracking of swimming motility

Bacterial suspensions were plated on microscope slides. The slides were imaged using KEYENCE BZ-X700 (Keyence, Osaka, Japan). The movies were then analyzed for bacterial swimming behavior using the video editing analysis software VW-H2 MA (Keyence, Osaka, Japan). Tracking data were subsequently processed using Microsoft Excel 2013 (Microsoft, Tokyo, Japan) to create x-y coordinate plots.

### Detection of flagellum

Bacteria were washed with PBS (pH 7.2) and stained with 1% phosphotungstic acid for 2 min. Flagella were imaged by electron microscopy.

### Cytotoxicity assay

Cytotoxicities against HeLa cells (n = 6/group, MOI = 10) were determined by measuring the activity of LDH released from damaged cells using a cytotoxicity assay kit (Cyto Tox 96; Promega KK, Tokyo, Japan).

### Mice

Five-week-old female C57BL/6 and BALB/c mice were purchased from Charles River Laboratories Japan (Atsugi, Japan). Except for the IVIS test, C57BL/6 mice were used for all experiments in our study. C57BL/6 mice were bred and maintained under specific pathogen–free conditions at Kitasato University, and BALB/c mice were bred and maintained under specific pathogen–free conditions at the Research Institute for Microbial Diseases, Osaka University.

### In vivo bioluminescent imaging

The plasmid pXen-13, which contains a bacterial luminescent gene cluster (*luxCDABE*), was transformed into *V. vulnificus* via electroporation. Electroporation was performed in a cuvette with a 0.2 cm electrode gap (Bio-Rad Laboratories, CA, USA). Stable transformants were selected on LB agar containing 100 µg/ml ampicillin. *V. vulnificus* was grown in LB medium supplemented with 100 µg/ml ampicillin with agitation (163 rpm) at 37°C. Overnight cultures (100 μl) were inoculated into 2 ml of fresh LB medium supplemented with 100 µg/ml ampicillin and incubated for 2 h. Bacteria were harvested, washed with PBS (pH 7.2) containing 0.1% gelatin, and resuspended in fresh LB medium. Then, 10^6^ CFU/mouse were s.c. inoculated in BALB/c mice. Luminescence signals emanating from *V. vulnificus* were imaged at defined time points using an IVIS 200 imaging system (Xenogen/PerkinElmer, MA, USA) with a 1 min exposure time. The total photons emitted were acquired using the Living Image software package. Mice were anesthetized in chambers containing 2.0% isoflurane inhalant (Pfizer, Tokyo, Japan).

### Histopathological examination

Infection sites excised from sacrificed mice were demineralized by immersing them in buffer solution containing 0.2 M EDTA-4Na and 1% formalin for 1 week, fixed in 10% buffered formalin for 1 day, embedded in paraffin, sliced into 3-µm sections, and stained with Hematoxylin-eosin (H&E). Image acquisition of the muscle tissue was performed using an inverted microscope (DM2500/Leica Microsystems, Tokyo, Japan) equipped with 20×/0.40 objective lenses.

Infected right caudal thighs excised from sacrificed mice were fixed in 4% paraformaldehyde for 24 h, cryoprotected for 1 h in 10% sucrose and for 12 h in 20% sucrose, embedded in FSC 22 Frozen Section Medium (Leica), and sliced into 3-µm sections. The sections were incubated with 1:1,000 rabbit anti-*V. vulnificus* antibodies for 1 h at room temperature. Subsequently, sections were washed twice in PBS for 5 min and incubated with PI and secondary antibodies (1:5,000 goat anti-rabbit IgG–Alexa Fluor 488) (Invitrogen, Tokyo, Japan) for 1 h at room temperature. Slides were washed again and coverslipped with Fluoromount (Diagnostic BioSystems, CA, USA). Image were obtained using an inverted microscope (DMI6000 B/Leica) at 490 and 556 nm excitation with 20×/0.40 CORR objective lenses. Images of each filter set were digitally merged using the Leica FW 4000 software.

### Bacterial counts in muscles and spleen

*V. vulnificus* was grown in LB medium supplemented with 100 µg/m*l* of rifampicin with agitation (163 rpm) at 37°C. Overnight cultures (100 μl) were inoculated into 2 ml of fresh LB medium and incubated for 2 h. Bacteria were harvested, washed with PBS (pH 7.2) containing 0.1% gelatine, and resuspended in fresh LB medium. Then, 10^6^ CFU/mouse were s.c. inoculated in mice. Infected mice were sacrificed at defined time points. The collected muscles or spleen were suspended in cold PBS containing 0.1% gelatine, homogenized for 5 s with a lab mixer IKA EUROSTAR digital (IKA Werke, Germany; 1,300 rpm), and centrifuged at 800 rpm for 5 min. The supernatants were plated at 10-fold serial dilutions in duplicate on LB agar containing 50 µg/ml rifampicin and incubated for 12 h at 37°C. *V. vulnificus* colonies were counted, and bacterial burden was determined by calculating the number of CFU/g.

### Evaluation of biomarkers following intramuscular damage

*V. vulnificus* was grown in LB medium supplemented with 100 µg/m*l* of rifampicin with agitation (163 rpm) at 37°C. Overnight cultures (100 μl) were inoculated into 2 ml of fresh LB medium and incubated for 2 h. Bacteria were harvested, washed with PBS (pH 7.2) containing 0.1% gelatine, and resuspended in fresh LB medium. Then, 10^6^ CFU/mouse were s.c. inoculated in mice. Infected mice were sacrificed at 9 hours post-infection. Whole-blood samples were collected in a syringe by cardiac puncture at multiple time points after infection and centrifuged at 1,200 g for 30 min, and sera were collected. Serum creatine kinase (CK), lactate dehydrogenase (LDH), and aspartate aminotransferase (AST) concentrations were evaluated using Dimension EXL with the LM Integrated Chemistry System (Siemens, Tokyo, Japan).

### Statistical analysis

Statistical analysis was performed using GraphPad Prism (GraphPad Software, CA, USA). Statistical differences between the two groups were analyzed using the Mann-Whitney *U* test or Fisher’s exact test. Survival curves were analyzed using the log-rank test. A P value less than 0.05 was considered significant, and Significance values are indicated as follow: *, *P* < 0.05; **, *P* < 0.01; ***, *P* < 0.001.

## Results

### In vitro virulence factors of V. vulnificus strains

**Figure 1.**
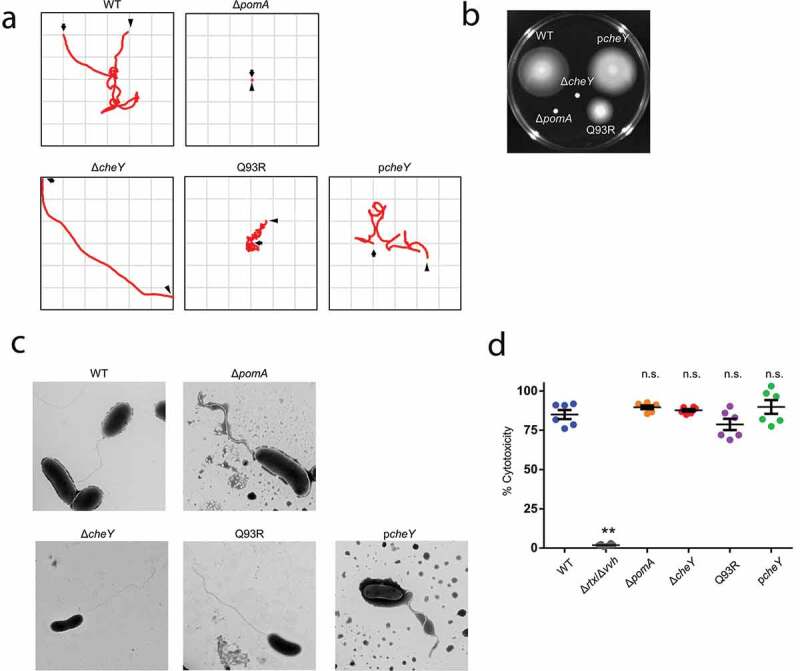
Phenotypic differences in swimming motility and swarming motility without affecting virulence factors.

We previously showed that the nonmotile Δ*pomA* mutant and nonchemotactic Δ*cheY* mutant could not produce fatal sepsis in a murine wound infection model [17]. In this study, we generated a point mutant of *cheYQ93R* (hereafter Q93R) for a more detailed investigation of the role of chemotaxis in the pathogenesis of NSTI. While the Δ*cheY* lacks the CheY activation, the point mutation was predicted to stabilize CheY phosphorylation (activation) [31]. Motility and chemotaxis are responsible for swimming, that is, individual movement in liquid, and for swarming, that is, multicellular movement on semi-solid surfaces. We first analyzed the defects in motility and chemotaxis of each strain by tracking swimming motility and by swarming plate assay. The analysis of swimming motility in the liquid media showed that WT moved by repeating smooth swimming and turning (tumbling), while Δ*pomA* never swam and turned (Figure 1(a), Movie S1, and S2). Δ*cheY* continued to swim straight and showed a wide net travel distance (Figure 1(a) and Movie S3). Q93R frequently turned and showed a narrow net travel distance in liquid media (Figure 1(a) and Movie S4). In a swarming assay using a semi-solid agar plate, WT showed a fully spread pattern, but Δ*pomA* and Δ*cheY* never spread (Figure 1(b)). The travel distance of Q93R decreased compared with WT (Figure 1(b)).

(A) Bacterial suspensions with LB broth were plated on microscope slides. Tracking data were subsequently processed to create x-y coordinate plots. The observation period, an arrow indicating the initial position, and an arrowhead indicating the final position are represented in the frame. (B) Bacteria were inoculated on LB plates containing 0.3% agar. Plates were incubated at 37°C and observed at 24 h post-inoculation. (C) Flagella of negative stained bacteria were imaged by electron microscopy. (D) Cytotoxicities against HeLa cells (n = 6/group, MOI = 10) were determined by measuring the activity of LDH released from damaged cells. Error bars indicate SEM. **P < 0.01; n.s., not significant (*P* > 0.05) compared with WT; Mann-Whitney *U*-test.

Our construction of the mutants, Δ*pomA*, Δ*cheY*, and Q93R, was predicted not to affect their flagellar structure, which contains major antigenic flagellin. These mutants were observed by electron microscopy, which confirmed that the flagella of Δ*pomA*, Δ*cheY*, and Q93R were the same as those of WT (Figure 1(c)). Also, the expression of cytotoxins was validated by LDH release assay. Two cytotoxins, VVH and RTX, are important factors for in vivo virulence of *V. vulnificus* [10,11], and the Δ*rtx*/Δ*vvh* lacking these two toxins completely lost its cytotoxicity against HeLa cells (Figure 1(d)). Since there was no significant difference in the cytotoxicity between all mutants and WT, the lack of motility or chemotaxis did not affect the known virulence factors (Figure 1(d)).

### Chemotactic motility to spread through soft tissues

**Figure 2.**
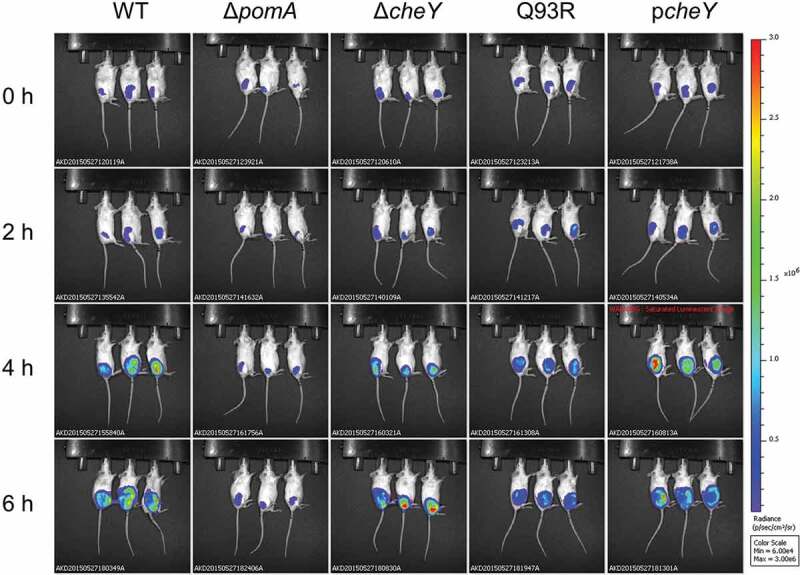
Chemotaxis-based spread through subcutaneous tissue. The luminescence signal of WT, Δ*pomA*, Δ*cheY*, Q93R, and p*cheY* detected by IVIS during 6 h time course.

Using a murine wound infection model of *V. vulnificus* we analyzed the effect of motility and chemotaxis on bacterial dynamics in soft tissues. To detect the infected bacteria by IVIS, each strain was transformed with a luciferase reporter plasmid pXen-*lux*. To rule out the possibility to lose the p-Xen lux and pACYC plasmids during infection, we analyzed the growth of all strains by both luminescence detection and plating diluted aliquots on agar plates with and without the antibiotics required to maintain the bacterial luciferase plasmid. There were no significant differences between the luminescence detection and growth in all strains with and without antibiotics (Fig. SI). These data indicate that the plasmid is maintained during the infection in vivo. BALB/c mice were injected subcutaneously at 1 × 10^6^ CFU. The bioluminescent signals from WT fully spread throughout the infected thigh at 6 hours post-infection, whereas nonmotile mutant Δ*pomA* stayed at the infected site (Figure 2). Signals from the nonchemotactic mutants, Δ*cheY* and Q93R, spread slowly compared with WT (Figure 2). These results showed that *V. vulnificus* spread through the soft-tissues within a short time, and that the chemotaxis of *V. vulnificus* was required to efficiently spread by both swimming in the liquid areas and swarming on the semi-solid parts of the soft-tissues.

### Chemotaxis-based invasion into muscle

**Figure 3.**
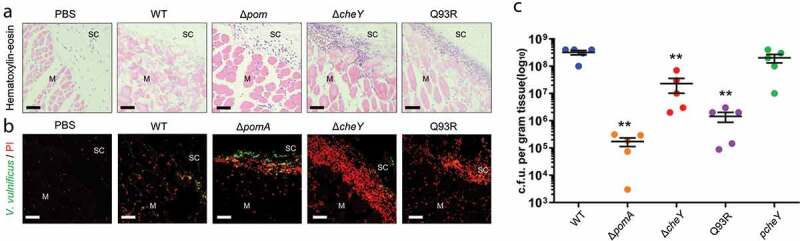
Chemotaxis-based invasion to muscle tissue.

To further investigate the role of the chemotaxis in bacterial spread, specifically in the invasion of muscle tissue from subcutaneous tissue, we performed histopathological examinations. H&E stained sections showed that muscle necrosis and tissue-infiltrating leukocytes in deep muscle tissue at the infection site in WT-inoculated mice (Figure 3(a)). In mice inoculated with Δ*cheY*, Q93R, and Δ*pomA*, muscle necrosis and leukocytes accumulation were observed on the fascial plane or in the superficial muscle tissue (Figure 3(a)). To clarify the localization of bacteria, frozen sections were stained with anti-*V. vulnificus* polyclonal antibodies and PI. WT were disseminated in soft-tissue, including deep muscle tissue, at the infection site, whereas all mutants existed in subcutaneous tissue and were colocalized with PI-positive cells (Figure 3(b)). The bacterial burden of WT in the muscle tissue was higher than in all mutants at 12 h post-infection (Figure 3(c)). These results demonstrated that chemotaxis is essential for efficient invasion of muscle at the local infection site by *V. vulnificus* in soft tissues.

(A and B) Histopathology analysis of right caudal thighs from infected mice. H&E (A) and anti-*V. vulnificus*/PI-stained (B) histopathology images of subcutaneous tissue and muscle tissue at infection sites in mice s.c.-inoculated with PBS, WT, Δ*pomA,* Δ*cheY*, and Q93R at 9 h post-infection. Scale bars, 50 μm. SC, subcutaneous tissue. M, muscle. (C) Bacterial burdens in the muscle tissue of mice s.c.-inoculated with WT, Δ*pomA*, Δ*cheY*, Q93R, and p*cheY* calculated as CFU/g at 12 h post-infection. Each symbol represents an individual mouse (*n* = 5/group). Error bars indicate SEM. **P < 0.01; n.s., not significant (*P* > 0.05) compared with WT; Mann-Whitney *U*-test.

### Chemotactic swimming accompanied with severe symptom

**Figure 4.**
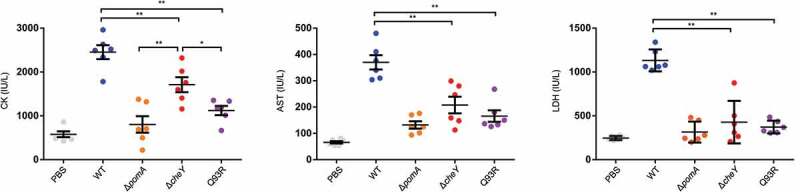
The tissue damage due to bacterial spread through soft tissue in *V. vulnificus* NSTI.

We determined the responsibility of the chemotaxis-based spread and invasion for producing the pathology of NSTI. Severity of tissue damage was evaluated by serum biochemical tests of CK, AST, and LDH. These enzymes are distributed in muscles when these cells are damaged. The levels of the enzymes in WT-inoculated mice were high. In contrast, the levels in Δ*pomA*-, Δ*cheY*- and Q93R-inoculated mice were significantly lower than that in WT-inoculated mice (Figure 4). These data coincided with the bacterial burden of the muscles (Figure 3(c)) and demonstrated that the motility and the chemotaxis of *V. vulnificus* during the infection contribute to the expansion of host tissue damage. Moreover, the serum CK levels of Δ*cheY*-inoculated mice were higher than those of Δ*pomA*- and Q93R-inoculated mice (Figure 4). This result suggested that the smooth swimming as seen in Δ*cheY* is a more important pathogenic factor for *V. vulnificus* than the frequent turning seen in Q93R (Figure 4).

Measurement of CK (left), LDH (center), and AST (right) levels in the sera of mice s.c.-inoculated with PBS, WT, Δ*pomA*, Δ*cheY*, and Q93R at 9 h post-infection. Each symbol represents an individual mouse (*n* = 6/group). Error bars indicate SEM. *P < 0.05, **P < 0.01; Mann-Whitney *U*-test.

### Chemotaxis-dependent bacterial invasion into the systemic circulation

**Figure 5.**
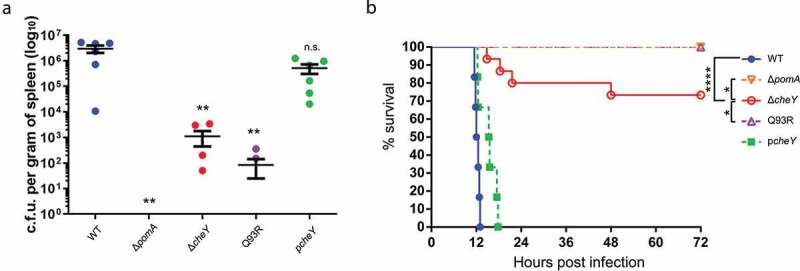
Invasion to the systemic circulation causing fatal outcomes.

The difference in severity between mice infected with Δ*cheY* and Q93R indicated the possibility that *V. vulnificus* invade deeper and distant tissues by smooth swimming, not only by spreading through the soft tissues. We hypothesized that the *V. vulnificus* invades the systemic circulation from the local infection site and investigated whether *V. vulnificus* could be detected in the spleen. Bacterial burdens of the spleen were detectable (n = 6 of 6) in the mice infected with WT and the *cheY*-complemented strain at 12 hours after infection, but Δ*pomA* was never detected (Figure 5(a) and Table 1). Bacterial burdens from the spleen of Δ*cheY*- and Q93R-infected mice were low (Figure 5(a)), and detection frequencies of Δ*cheY* and Q93R were also low compared with WT (Table 1, *P* = 0.0014 and *P* < 0.0001, respectively; Fisher’s exact test). These data indicated that motility and chemotaxis were involved in the invasion into the systemic circulation. However, there was no significant difference in the detection frequency between Δ*cheY* and Q93R at 12 h post-infection (*P* = 0.3202; Fisher’s exact test). Therefore, we analyzed the detection frequency of Δ*cheY* and Q93R at 24 h post-infection. It was revealed that Δ*cheY* was detected from spleen at a higher frequency compared with Q93R at 24 h (Table 1, *P* = 0.0076; Fisher’s exact test). At that time, all mice infected with WT had already died. The percent survival of mice infected with Δ*cheY* was higher than that with WT and lower than that with Q93R or Δ*pomA* (Figure 5(b)). Thus, the frequency of detection from the spleen correlated not only with the tissue damage but also with the lethality in mice (Figures 4 and 5(b)). Smooth swimming was important for invasion into the systemic circulation resulting in lethal NSTI.

**Table 1.** Phenotypes in the motility of Δ*pomA*, Δ*cheY*, and Q93R, the number of detections from spleen at the two-time points, and the number of dead mice after infection.

Strains	swimming phenotype (in liquid media)	swarming phenotype (on semi-solid media)	Systemic Dissemination 12 h.p.i.	Systemic Dissemination 24 h.p.i.	Death
Δ*pomA*	non-swimming	non-swarming	0/6	0/12	0/12
Δ*cheY*	smooth bias	non-swarming	4/6	10/12	4/12
Q93R	turn (tumble) bias	intermediate phenotype compared to WT	2/6	0/12	0/12

Abbreviation: h.p.i., hours post-infection

(A) Bacterial burdens in the spleen of mice s.c.-inoculated with WT, Δ*pomA*, Δ*cheY*, Q93R, and p*cheY* calculated as CFU/g at 12 h post-infection. Each symbol represents an individual mouse (*n* = 6/group). Error bars indicate SEM. **P < 0.01; n.s., not significant (*P* > 0.05) compared with WT; Mann-Whitney *U*-test. (B) Kaplan-Meier survival curves for mice inoculated s.c. with WT (*n* = 6), Δ*pomA* (*n* = 12), Δ*cheY* (*n* = 12), Q93R (*n* = 12), and p*cheY* (*n* = 6) and monitored for 72 h. *P < 0.05, ****P < 0.0001; log-rank test.

## Discussion

Despite the fact that lesions in NSTI progress dramatically from the epidermis through complex tissues such as dermis, hypodermis, subcutaneous fat, fascia, muscle, and into the systemic circulation [1–4], the pathogenic factors that cause the rapid expansion of these infections in patients have not been elucidated. We showed here for the first time that the chemotaxis of *V. vulnificus* was essential for rapid bacterial spread from the infection site to a deep layer of muscle and systemic circulation through complex tissues by appropriately switching from smooth swimming to turn (tumble) and from swimming to swarming in wound infection.

In liquid media, Δ*cheY* swam straight over a wide range, and Q93R frequently turned in a narrow range (Figure 1(a)). On the other hand, in a swarming assay on soft agar plate, Δ*cheY* could not spread, whereas Q93R could (Figure 1(b)). Thus, the Δ*cheY* phenotype spread by means of smooth swimming in the liquid part and the Q93R phenotype spread by swarming on semi-solid surfaces during the process of soft-tissue infection in this murine model (Figure 2). Necrotizing fasciitis is known to expand rapidly along the fascia, suggesting that *V. vulnificus* is swarming on the fascia, which is a semi-solid part of soft tissues, based on chemotaxis. However, liquid parts are uncommon in soft tissues as compared with stomach, intestines, bladder, and systemic circulation. Miyoshi et al showed that *V. vulnificus* induces edematous skin lesions as a result of increased vascular permeability by metalloproteinase VvpE [15]. These facts suggested that *V. vulnificus* may create a liquid area where it can efficiently spread through the soft tissues by swimming. Staying at the local infection site will cause nutrient competition and depletion, which are not desirable for bacterial proliferation. To avoid this competition, bacteria have chemotaxis that enables them to respond to changes in the attractant gradient, travel, and efficiently acquire nutrients.

We previously showed that Δ*pomA* and Δ*cheY* were attenuated mutants that could not proliferate enough to be detected from spleen [17]. The detailed study of these mutants here revealed that their ability to invade the muscle and the systemic circulation was reduced (Figures 3(c) and 5(a)). These findings indicate that motility and chemotaxis are required for efficient invasion. In addition, smooth swimming, as seen in Δ*cheY*, was consistently associated with higher virulence than turning and swarming, as seen in Q93R. The CK level, the detection frequency in the spleen, and the lethality of mice infected with the Δ*cheY* were higher than in Q93R (Figures 4, 5(a), and 5(b)). These data suggest that swimming in liquid parts of soft tissues is more essential for pathogenicity than the swarming on the semi-solid areas during the infection. Indeed, Butler and Camilli showed that Δ*cheY* of *V. cholerae* was more pathogenic than its WT in the intestinal tract, which is one of the host’s most liquid parts [28]. It was concluded that smooth swimming in the liquid was important as it allowed the agent to move over a wider range [25,28]. Besides being the most major liquid part of the body, the proliferation of pathogens in the systemic circulation is directly linked to lethal sepsis. In particular, the systemic spread of *V. vulnificus* by chemotactic swimming was shown to be lethal in the wound infection model.

All strains used for the infection in this study showed cytotoxicity in vitro (Figure 1(d)), and tissue damages of all infected mice were high compared with the PBS-inoculated control (Figure 4). This means that the mutants retained cytotoxins VVH and RtxA even though lacking motility or chemotaxis. RtxA is indispensable in establishing *V. vulnificus* infection by killing neutrophils [10]. By the secretion of RtxA, *V. vulnificus* survived at the site of infection without being killed by neutrophils during the IVIS observation period (Figure 2). Nevertheless, tissue damages in all mice infected with Δ*pomA*, Δ*cheY*, and Q93R were much lower than that of the WT (Figure 4). These results indicate that the cytotoxic activity of *V. vulnificus* will not occur in distant tissue in the absence of chemotaxis, and that lesions occur only where the bacteria are present. These findings taken together show that chemotaxis is the most important factor for establishing the rapid expansion of lesions as a clinical feature of *V. vulnificus* NSTI.

This is the first study to report chemotaxis as one of the most critical factors for the evolution of NSTI, by analyzing the host-pathogen dynamics. These findings could yield important clinical benefits in the determination of the degree of surgical debridement, which has a significant impact on patient quality of life. This study also suggests that chemotaxis is an attractive target for the development of novel prevention methods against the progression to necrosis and sepsis. One future research topic will be whether limiting chemoattractants can inhibit bacterial migration to more appropriate sites for proliferation during NSTI.

## Supplementary Material

Supplemental Material
