# Supplementary material for: Chemotactic invasion in deep soft tissue by Vibrio vulnificus is essential for the progression of necrotic lesions
Source: Virulence. 2020 Jun 27;11(1):840–8. doi: 10.1080/21505594.2020.1782707 (PMC7550010; doi:10.1080/21505594.2020.1782707)
Supplement: Supplemental Material [file KVIR_A_1782707_SM5186.docx]

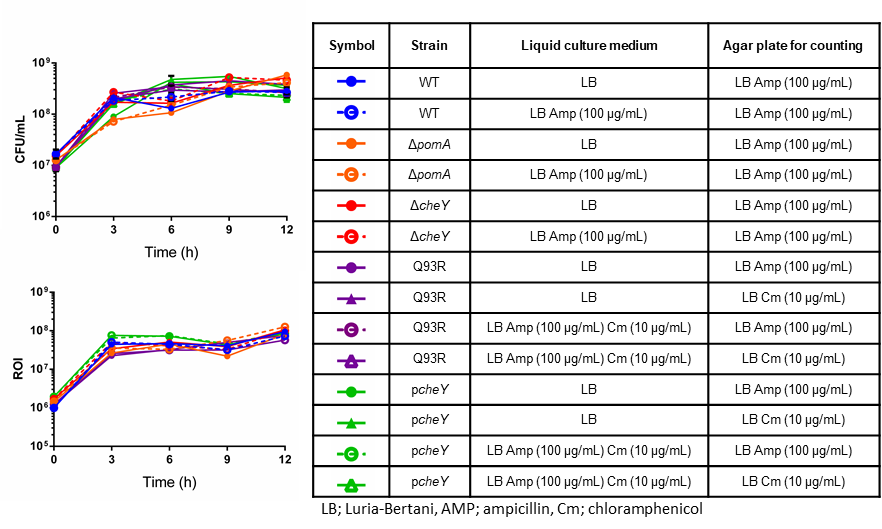
Figure S1. Growth curves of strains with and without antibiotics. WT, Δ*pomA*, and Δ*cheY* were cultured aerobically in LB broth supplemented with ampicillin (100 µg/mL) at 37°C for overnight. Q93R and p*cheY* were cultured aerobically in LB broth supplemented with ampicillin (100 µg/mL) and chloramphenicol (10 µg/mL) at 37°C for overnight. Bacterial suspensions were adjusted to an optical density at 600 nm of 0.1 and inoculated to LB broth with and without antibiotics. At each time point, CFU/ML was detected by plating diluted aliquots on agar plates, and bacterial luminescence was detected by in vivo imaging system as ROI. Error bars indicate SEM.
